# Impact of Renal Function on the Surgical Outcomes of Displaced Femoral Neck Fracture in Elderly Patients

**DOI:** 10.3390/jcm8081149

**Published:** 2019-08-01

**Authors:** Hyuck Min Kwon, Suhan Lim, Ick-Hwan Yang, Woo-Suk Lee, Byeong Hun Jeon, Kwan Kyu Park

**Affiliations:** Department of Orthopedic Surgery, College of Medicine, Yonsei University, Seoul 03722, Korea

**Keywords:** hemiarthroplasty, geriatric femoral neck fracture, chronic kidney disease, estimated glomerular filtration rate

## Abstract

Background: The aim of this study was to investigate the relationship between estimated glomerular filtration rate (eGFR) and outcomes of bipolar hemiarthroplasty for femoral neck fracture in elderly patients, and to compare postoperative complications and mortality among groups according to eGFR. Methods: A total of 181 patients who underwent bipolar hemiarthroplasty for displaced femoral neck fracture were divided into three groups according to eGFR. Data were retrospectively analyzed. Group 1 had 96 patients with eGFR greater than or equal to 60 mL/min/1.73 m^2^; Group 2 had 54 patients with eGFR greater than or equal to 30 mL/min/1.73 m^2^ and lower than 60 mL/min/1.73 m^2^; and Group 3 had 31 patients with eGFR lower than 30 mL/min/1.73 m^2^. Postoperative complications and mortality were compared between groups at a minimum 2-year follow-up. Results: Patients in Group 3 had the longest hospital stay of the three groups (*p* = 0.001). The rates of medical complications did not differ significantly among groups. However, Group 2 and 3 had higher rates of surgical complications (*p* = 0.001) and mortality (*p* = 0.043) than Group 1. Severe renal impairment was associated with increased risk of postoperative complications compared to mild renal impairment (odds ratio (95% confidence interval) = 4.33 (1.32–13.19), *p* = 0.015). Conclusion: Patients with moderate or severe decreased eGFR associated with chronic kidney disease (CKD) could have higher postoperative complications and mortality after bipolar hemiarthroplasty compared to patients with CKD stage 1 or 2.

## 1. Introduction

Several studies have reported excellent outcomes of bipolar hemiarthroplasty in terms of early recovery and ambulation for displaced femoral neck fracture in elderly patients [1,2,3]. Many orthopedic surgeons prefer bipolar hemiarthroplasty for displaced femoral neck fracture over total hip arthroplasty or internal fixation in elderly patients with a low degree of physical activity and decreased cognitive function [4,5]. However, mortality ranges from 15% to 40% at 1 year after femoral neck fracture in elderly patients, so it is important to understand how surgical treatment and postoperative care may affect mortality and comorbidities [6,7,8,9].

Patients with chronic kidney disease (CKD), defined as a decreased kidney function (estimated glomerular filtration rate; eGFR) or the presence of kidney damage, may develop metabolic bone diseases such as osteomalacia with low bone turnover, which could increase the risk of fracture due to poor bone quality and muscle weakness [10]. CKD can be categorized into five stages according to eGFR: Stage 1 (normal, eGFR > 90 mL/min/1.73 m^2^ with other evidence of chronic kidney damage); Stage 2 (mild impairment, 60 < eGFR < 89 mL/min/1.73 m^2^ with other evidence of chronic kidney damage); Stage 3 (moderate impairment, 30 < eGFR < 59 mL/min/1.73 m^2^); Stage 4 (severe impairment, 15 < eGFR < 29 mL/min/1.73 m^2^); and Stage 5 (established renal failure, eGFR < 15 mL/min/1.73 m^2^ or on dialysis) [11,12]. Patients with end-stage renal disease (ESRD) have an increased risk of hip fracture even with minimal trauma, and they have a high risk of mortality and perioperative complications when operative treatment is performed because they often have accompanying medical problems, such as chronic anemia, cardiovascular disease, cerebrovascular disease, and diabetes [13,14,15]. Previous studies have demonstrated increased surgical complications for ESRD patients with femoral neck fracture, but the relationship between decreased eGFR and outcomes of bipolar hemiarthroplasty for displaced femoral neck fracture has not yet been clearly investigated [16]. This staging system can be used to assess the impact of decreased eGFR on clinical outcomes of bipolar hemiarthroplasty with displaced femoral neck fracture in elderly patients.

We investigated the relationship between decreased eGFR and outcomes of bipolar hemiarthroplasty for displaced femoral neck fracture in elderly patients and identified factors associated with increased surgical and medical postoperative complications and mortality. We hypothesized that elderly patients with moderate or severe renal impairment would have increased complications, mortality, and morbidity compared to patients with normal or mild impairment of renal function.

## 2. Materials and Methods

### 2.1. Patient Recruitment and Study Design

This study was approved by the Institutional Review Board of Severance Hospital (4-2018-1112, approved on 17 January 2019). The study included 181 patients older than 60 years who underwent bipolar hemiarthroplasty for displaced femoral neck fracture after a slip down injury between March 2015 and December 2016 and who completed at least 2 years of follow-up. The inclusion criteria were independent living status, independent walking before injury, and absence of severe cognitive dysfunction. The exclusion criteria were pathological fracture, open fracture, fracture with hip dislocation, previous hip disease such as osteoarthritis or rheumatoid arthritis, and previous hip operation. The study population included 56 men and 125 women with a mean age of 77 years (range, 61 to 94 years). After the diagnosis of displaced femoral neck fracture with radiologic examination, surgical treatment was performed within 48 h of the injury.

### 2.2. Surgical Interventions

All bipolar hemiarthroplasties for displaced femoral neck fracture were performed by a single experienced surgeon who used the same techniques for all patients. The modified Gibson’s posterolateral approach was used with the patient in the lateral decubitus position. The femoral stem Accolade^®^ II (Stryker Orthopaedics, Mahwah, NJ, USA) and bipolar head (UHR, Stryker Orthopaedics, Mahwah, NJ, USA) were used in all operations with a non-cementing technique. All patients were treated with low-molecular-weight heparin preoperatively and for 5 days post-operatively. They also used antithrombotic stockings. Prophylactic antibiotics with a first-generation cephalosporin were given pre-operatively and during the first 24 h after surgery. All patients were treated with the same postoperative protocols. Sitting was encouraged on the first operation day, as well as immediate ambulation with full weight bearing with the aid of a walker in a tolerable range. The patients were educated about precautions they should take to avoid dislocation.

### 2.3. Renal Function Assessment

To determine renal function, we calculated the estimated glomerular filtration rate (eGFR) by the abbreviated Modification of Diet in Renal Disease (MDRD) equation (eGFR in mL/min/1.73 m^2^) = 186 × (serum creatinine (mg/dL))^−1.154^ × (age (year))^−0.203^ × (0.742 if the patient was female) [12]. We used the National Kidney Foundation Kidney Disease Outcomes Quality Initiative (NKF-K/DOQI) guidelines to diagnose and define CKD [17]. Patients were divided into three groups: Group 1 had 96 patients with eGFR greater than or equal to 60 mL/min/1.73 m^2^; Group 2 had 54 patients with eGFR greater than or equal to 30 mL/min/1.73 m^2^ and lower than 60 mL/min/1.73 m^2^; and Group 3 had 31 patients with eGFR lower than 30 mL/min/1.73 m^2^. All patients met the inclusion/exclusion criteria.

### 2.4. Clinical Outcomes Assessment

All clinical information was collected using pre-designed datasheets in the outpatient clinic pre- and postoperatively and was maintained in our database by an independent investigator. The clinical information included demographic data, preoperative clinical status, and postoperative outcomes. The patients were interviewed about their ambulation and activities of daily living before injury using the American Society of Anesthesiologists (ASA) classification information [18]. Duration of surgery and length of hospital stay were recorded. Peri-operative medical complications at postoperative 3 months were recorded. Complications related to surgery were recorded for a minimum postoperative 2 years. Mortality during the follow-up period (minimum 2 years) was also recorded.

### 2.5. Statistical Analysis

All statistical analyses were performed with SPSS version 20 for Windows (SPSS Inc., Chicago, IL, USA), and *p* values < 0.05 were considered statistically significant. Clinical outcomes were compared among the three groups. Analysis of variance (ANOVA) and Chi-squared tests were used to compare groups and logistic regression analysis was performed. Odds ratios (ORs) with 95% confidence intervals (CIs) were calculated to identify factors associated with postoperative complications.

## 3. Results

The median (interquatile range, IQR) eGFR of Groups 1, 2, and 3 were 85 (78–90), 44 (37–52), and 9 (6–17), respectively. There were no significant differences in age, sex, BMI or operation time among the three groups (Table 1). However, Group 3 (GFR < 30 mL/min/1.73 m^2^) had lower hemoglobin levels (*p* = 0.001, 10.1 ± 1.1 g/dL) than Group 1 (12.7 ± 1.7 g/dL) or Group 2 (11.4 ± 2.4 g/dL) (*p* = 0.001). There were higher incidences of hypertension (*p* = 0.011), coronary artery disease (*p* = 0.001), and cerebrovascular disease (*p* = 0.001) in Group 3 than Group 1 or Group 2. Patients in Group 3 had the longest hospital stay of the three groups (22.6 ± 37.5 days, *p* = 0.001).

Table 2 summarizes all surgical and medical complications and mortality associated with bipolar hemiarthroplasty for displaced femoral neck fracture at a minimum follow up of 2 years. The 2-year mortality of all subjects was 16%. Group 1 had six cases (6.3%) of surgical complications, five cases (5.2%) of medical complications, and 12 cases of 2-year mortality (12.5%). Group 2 had seven cases (13%) of surgical complications, six cases (11.1%) of medical complications, and eight cases (14.8%) of 2-year mortality. Group 3 had seven cases (22.6%) of surgical complications, six cases (19.4%) of medical complications, and nine cases (29%) of 2-year mortality. All medical complications in the three groups occurred within the first month after surgery. All surgical complications in the three groups occurred after postoperative 1 month (1–39 months). The rates of medical complications did not differ significantly among the three groups. However, the rate of surgical complications and 2-year mortality were highest in Group 3 (respectively, *p* = 0.001 and *p* = 0.043). The details of surgical and medical complications of bipolar hemiarthroplasty are summarized in Table 3 and Table 4.

In the analysis of the risk factors for surgical and medical complications, high odds ratios (OR) were found in Group 2 or Group 3 (Group 2: OR (95% CI) = 2.45 (1.01–5.94), *p* = 0.047/Group 3: OR (95% CI) = 5.58 (2.16–14.33), *p* = 0.001 versus Group 1 in univariate analyses (Table 5). Logistic regression analysis revealed a high odds ratio (OR) in Group 3 (OR (95% CI) = 4.3 (1.32–14.19), *p* = 0.015 versus Group 1) with a multivariate model that included age, sex, BMI, hemoglobin, and ASA classification, and all of the variables listed in Table 5. Univariate analysis showed that moderate and severe renal impairment (Group 2 and Group 3) might be risk factors for increased surgical and medical complications; however, none of the evaluated factors were associated with complications in multivariate analysis.

## 4. Discussion

This study investigated whether renal function affects the surgical outcomes of bipolar hemiarthroplasty for displaced femoral neck fracture in elderly patients. The most important finding of the present study was that patients with severe decreased renal function associated with CKD stage 4 or 5 had higher postoperative complication rates and mortality than patients with CKD stage 1 or 2, even though peri-operative medical complications of surgery did not differ significantly among the groups. In addition, patients with moderate decreased renal function with CKD stage 3 had also higher postoperative complication rates and mortality. This study demonstrated that renal function as measured by eGFR could be a factor influencing the likelihood of complications of bipolar hemiarthroplasty for displaced femoral neck fracture in elderly patients. Moreover, elderly patients with decreased renal function would be at risk of further postoperative complications due to comorbidities associated with renal impairment. Previous studies have suggested that ESRD patients who undergo surgery for displaced femoral neck fracture have high complication rates and increased mortality [19]. We further demonstrated that moderate decreased renal function as well as severe decreased renal function could influence the surgical outcomes of bipolar hemiarthroplasty for displaced femoral neck fracture in terms of postoperative complications and mortality.

In our study, patients in Group 3 (eGFR lower than 30 mL/min/1.73 m^2^) had low hemoglobin levels. This may have been due to insufficiency of erythropoietin (EPO) because healthy kidneys produce EPO. Additionally, malnutrition in association with CKD and chronic blood loss in dialysis might also lead to a low level of hemoglobin. This chronic anemia may have had a negative effect on the surgical outcomes of bipolar hemiarthroplasty. Moreover, patients in Group 3 had more comorbidities, such as hypertension, coronary artery disease, and cerebrovascular disease. These conditions could have increased the length of hospital stay and led to higher 2-year mortality in Group 3 than in the other groups. The longer length of hospital stay could also lead to increased nosocomial infections such as pneumonia, as well as early and late periprosthetic joint infection [20]. In addition, patients with decreased eGFR may experience sudden drug-induced renal toxicity due to the use of prophylactic IV antibiotics and anti-inflammatory analgesics during hospitalization for surgery [21]. Because we demonstrated that the eGFR group was a factor affecting postoperative medical and surgical complications of bipolar hemiarthroplasty, it is possible that eGFR might be an important indicator of the postoperative outcomes of bipolar hemiarthroplasty with displaced femoral neck fracture. Since renal function represented by eGFR is important in the prediction of postoperative complications of bipolar hemiarthroplasty in elderly patients, sufficient preoperative evaluation of renal function and a strictly limited fluid regimen should occur during hospitalization to reduce postoperative mortality and morbidity.

Decreased renal function of patients who undergo bipolar hemiarthroplasty for geriatric displaced femoral neck fracture can also result in other aspects of poor bone health. Osteoporosis and renal osteodystrophy associated with CKD might cause poor bone metabolism and increased fracture incidence, potentially contributing to postoperative periprosthetic fracture or implant failure [22]. Additionally, patients with CKD could have vitamin D deficiency and serum calcium-phosphate imbalance caused by poor bone health [23]. For these reasons, we confirmed that patients with decreased renal function would have high surgical complication rates of bipolar hemiarthroplasty due to poor bone health at a minimum 2-year follow-up. However, this study found no significant difference in medical complications according to renal function, perhaps because bipolar hemiarthroplasty for displaced femoral neck fracture was generally performed in elderly patients with a low degree of physical activity [24].

Bipolar hemiarthroplasty may be more appropriate than total hip arthroplasty for elderly patients with decreased renal function who experience displaced femoral neck fracture. In particular, patients with CKD stage 4 or 5 would have a lower physical activity level than those with normal renal function of the same sex and age [25,26,27], so bipolar hemiarthroplasty could be indicated for these patients even if they are relatively young. Although bipolar hemiarthroplasty has been considered a good surgical treatment for displaced femoral neck fracture in elderly patients, 1-year mortality rates have been reported at up to 40% [6,8]. Because most deaths are due to perioperative medical comorbidities, such as postoperative pneumonia and coronary or cerebral vascular disease, mortality is expected to be higher in elderly patients with decreased renal function [28]. In addition, postoperative ambulation and rehabilitation of patients with decreased renal function is often delayed because of poor bone quality and muscle power. These factors could lead to a poor prognosis of bipolar hemiarthroplasty for patients with decreased renal function, especially ESRD patients who require dialysis. Thus, it is important to understand the risk of complications and mortality of bipolar hemiarthroplasty in elderly patients with decreased renal function.

There were several limitations to our study. First, the study included relatively few patients. Although the sample size of 181 patients was modest, only 31 patients with decreased eGFR of stage 4 or 5 were included. Moreover, since the number of patients in each group was too small to divide the patients into five groups, CKD stages 1 and 2 were defined as the mild decreased~normal kidney function group, which could affect the conclusions. For several patients with severely decreased renal function, postoperative follow-up was discontinued due to the patient’s death or severe comorbidities. Therefore, we conclude that decreased eGFR may affect surgical outcomes of femoral neck fracture, and further survivorship analysis using this as a risk factor and registration of more subjects with severely decreased renal function is needed. Second, the clinical data were not collected prospectively; rather, retrospective analysis of radiologic data and medical records was performed. Thus, we were limited to the data that were documented. Finally, information about osteoporosis and postoperative physical function, such as the lower extremity measure (LEM) score, were not documented, so we could not evaluate the effect of decreased eGFR on osteoporosis and clinical outcomes of bipolar hemiarthroplasty [29]. Nevertheless, this was the first study comparing complications and mortality after bipolar hemiarthroplasty among groups that differed in eGFR.

## 5. Conclusions

Patients with moderate or severe decreased eGFR associated with CKD could have higher postoperative complications and mortality after bipolar hemiarthroplasty compared to patients with CKD stage 1 or 2. Since bipolar hemiarthroplasty for displaced femoral neck fracture for elderly patients with decreased eGFR was predictive of a poor prognosis after surgery, proper pre- and postoperative evaluation and special attention are required.

## Figures and Tables

**Table 1:** Demographic data.

Parameter	Total (*n* = 181)	Group 1 (*n* = 96, eGFR ≥ 60)	Group 2 (*n* = 54, 60 > eGFR ≥ 30)	Group 3 (*n* = 31, eGFR < 30)	*p*
Age in years (range)	77.1 ± 8.1 (61–94)	79.1 ± 7.7 (61–94)	78.2 ± 7.9 (64–94)	74.3 ± 8.2 (63–91)	0.078
Sex (male/female)	56:125	29:67	18:36	9:22	0.412
BMI (kg/m^2^)	22.9 ± 5.1	23.1 ± 4.7	23.3 ± 4.1	22.4 ± 3.6	0.082
Hemoglobin (g/dL)	11.8 ± 1.9	12.7 ± 1.7	11.4 ± 2.4	10.1 ± 1.1	0.001
Medical history					
Hypertension	123 (67.9%)	66 (68.8%)	38 (70.3%)	25 (80.6%)	0.011
Diabetes mellitus	91 (50.3%)	47 (49%)	27 (50%)	19 (61.3%)	0.426
Coronary artery disease	36 (19.9%)	19 (19.8%)	17 (31.5%)	12 (38.7%)	0.001
Cerebrovascular disease	29 (16%)	14 (14.6%)	11 (22.2%)	10 (32.3%)	0.001
ASA classification					
I	0 (0%)	0 (0%)	0 (0%)	0 (0%)	
II	44 (24.3%)	40 (41.7%)	4 (7.4%)	0 (0%)	
III	118 (65.2%)	56 (58.3%)	44 (81.5%)	18 (58.1%)	
IV	19 (10.5%)	0 (0%)	6 (11.1%)	13 (41.9%)	
Operation time (mins)	92.5 ± 46.4	93 ± 41.7	89.1 ± 42.7	89.8 ± 48.4	0.725
Hospital stay (days)	11.8 ± 15.9	9.1 ± 8.7	10.4 ± 4.4	22.6 ± 37.5	0.001

eGFR, estimated glomerular filtration rate.

**Table 2:** Postoperative complications and mortality.

Complications	Total (*n* = 181)	Group 1 (*n* = 96, eGFR ≥ 60)	Group 2 (*n* = 54, 60 > eGFR ≥ 30)	Group 3 (*n* = 31, eGFR < 30)	*p*
Surgical complications	20 (11%)	6 (6.3%)	7 (13%)	7 (22.6%)	<0.001
Medical complications	13 (7.2%)	5 (5.2%)	6 (11.1%)	6 (19.4%)	0.056
Total complications	33 (18.2%)	11 (10.5%)	13 (24.1%)	13 (42%)	<0.001
2-year mortality	29 (16%)	12 (12.5%)	8 (14.8%)	9 (29%)	0.043

**Table 3:** Surgical complications.

Case	eGFR Group	Age	Sex	Complications	Time (Postoperative Months)	Treatment
1	1	76	M	Periprosthetic fracture	1	Operation
2	1	74	M	Dislocation	3	Closed reduction
3	1	76	F	Periprosthetic fracture	6	Operation
4	1	80	F	Periprosthetic fracture	6	Conservative treatment
5	1	77	F	Subsidence of prosthesis	12	Operation
6	1	73	F	Myositis ossification	10	Conservative treatment
7	2	73	F	Superficial wound infection	3	Conservative treatment
8	2	81	M	Periprosthetic fracture	15	Operation
9	2	70	M	Periprosthetic fracture	12	Operation
10	2	68	F	Periprosthetic fracture	18	Conservative treatment
11	2	77	F	Periprosthetic fracture	7	Operation
12	2	75	F	Subsidence of prosthesis	12	Operation
13	2	72	F	Subsidence of prosthesis	14	Operation
14	3	91	M	Dislocation	3	Closed reduction
15	3	75	F	Subsidence of prosthesis	8	Conservative treatment
16	3	75	F	Periprosthetic fracture	18	Operation
17	3	76	F	Periprosthetic fracture	1	Conservative treatment
18	3	77	F	Periprosthetic fracture	3	Operation
19	3	71	F	Periprosthetic fracture	39	Conservative treatment
20	3	78	F	Subsidence of prosthesis	8	Operation

M, male; F, female.

**Table 4:** Medical complications.

Case	eGFR Group	Age (years)	Sex	Complications	Time (Postoperative Days)
1	1	88	F	Lobar pneumonia	14
2	1	87	F	Pulmonary embolism	3
3	1	92	M	Aspiration pneumonia	12
4	1	77	F	Lobar pneumonia	7
5	1	86	M	Lobar pneumonia	5
6	2	88	F	Aspiration pneumonia	2
7	2	76	F	Myocardial ischemia	3
8	2	71	F	Lobar pneumonia	3
9	2	79	F	Pulmonary embolism	7
10	2	84	F	Lobar pneumonia	5
11	2	81	F	Lobar pneumonia	12
12	3	76	F	Aspiration pneumonia	3
13	3	78	F	Myocardial ischemia	5
14	3	84	M	Lobar pneumonia	7
15	3	81	F	Lobar pneumonia	11
16	3	71	F	Cerebrovascular accident	2
17	3	71	F	Lobar pneumonia	5

**Table 5:** Risk factors for surgical and medical complications.

Factor	Group	Univariate		Multivariate	
		OR (95% CI)	*p* Value	OR (95% CI)	*p* Value
Age		1 (0.93–1.07)	0.564		
Sex		1.13 (0.38–3.4)	0.826		
BMI		0.87 (0.76–1.01)	0.059		
Hemoglobin		0.76 (0.57–1.02)	0.071		
eGFR group	1	Ref.			
	2	2.45 (1.01–5.94)	0.047		
	3	5.58 (2.16–14.43)	0.001	4.33 (1.32–13.19)	0.015
Medical history	HTN	0.62 (0.22–1.81)	0.385		
	DM	0.66 (0.24–1.81)	0.417		
	CAD	1.3 (0.39–4.33)	0.67		
	CVD	3.34 (1.52–8.4)	0.166		
ASA classification	I				
	II	Ref.			
	III	6.38 (0.81–50.20)	0.079		
	IV	8.50 (0.68–105.75)	0.096		
Fracture type (Garden stage)	I	Ref.			
	II	0	0.999		
	III	3.89 (0.41–37.19)	0.24		
	IV	2.05 (0.25–17.02)	0.507		
Operation time		1 (0.99–1.01)	0.441		
Hospital stay		1.05 (1.01–1.09)	0.067		
